# Determinants of diabetic retinopathy in Southwest Ethiopia: a facility-based case-control study

**DOI:** 10.1186/s12889-020-08652-2

**Published:** 2020-04-15

**Authors:** Dugasa Garoma, Hailu Merga, Desta Hiko

**Affiliations:** 1 Nekemte College of Health Sciences, Nekemte, Ethiopia; 2 Department of Epidemiology, Institute of Health, Jimma University, Jimma, Ethiopia

**Keywords:** Diabetic retinopathy, Determinants, Case control study Southwest Ethiopia

## Abstract

**Background:**

Diabetic Retinopathy is one of the serious complications patients’ diabetic patients suffer from. Little is known about which risk factors are associated with this complication. The aim of this study was therefore to identify determinants of Diabetic Retinopathy in Jimma University Medical Center.

**Methods:**

A facility-based case-control study was conducted. Cases were Diabetic patients with diabetic retinopathy and who were on follow up at the Jimma University Medical Center while controls were Diabetic patients but free of diabetic retinopathy and who were on follow up at the Jimma University Medical Center. Cases and controls were identified and 311 of them were recruited using systematic random sampling. Data were entered into the Epi-Data version 4.1 and analyzed using SPSS Version 20. Binary Logistic regression analysis was conducted to identify determinants of diabetic retinopathy.

**Result:**

A total of 106 cases and 205 controls diabetic participated in the study. Being ≥60 years of age (AOR = 5.04,95%CI: 1.83,13.87),being illiterate (AOR = 7.17, 95% CI: 2.61,19.7), poor adherence to medication (AOR =3: 95% CI: 1.29,6.95),having high systolic blood pressure (AOR = 3.38:95% CI: 1.26,9.05), having family history of Diabetes Mellitus (AOR = 3.95: 95% CI: 1.64,9.54), having other micro vascular complications (AOR = 3.76,95% CI: 1.33,10.66), poor glycemic control (AOR = 9.08, 95%CI: 3.7,22.29), poor cholesterol control (AOR = 0.21, 95%CI: 0.08,0.51) and being anaemic (AOR = 2.8, 95%CI: 1.05,7.47) were the independent determinants of diabetic retinopathy.

**Conclusion:**

This study found that poor adherence to medication, being at the age of 60 years and above, being illiterate patients, having high systolic blood pressure, having a family history of Diabetes Mellitus, having other micro vascular complication, poor glycemic control, poor cholesterol control and being anemic patient were the independent determinants of diabetic retinopathy. Therefore, more attention should be given to older age and illiterate patients. Giving more emphasis for patients poorly adhered to anti-diabetic medications and giving advice for diabetic patients with high systolic blood pressure to follow their blood pressure regularly are also vital. Diabetic patients should also control their Blood sugar and blood cholesterol levels to prevent diabetic retinopathy or reduce its further complications.

## Background

Diabetic retinopathy (DR) is a neurovascular complication of both type1 and type 2 diabetes, which strongly correlates to both the duration of diabetes and the level of glycemic control. It is the major long-term complication of diabetes and the main cause of vision impairment and vision loss. Diabetic retinopathy is a primary cause of visual injury in working-age adults [1, 2].

Diabetic retinopathy is the specific micro-vascular complication of Diabetes Mellitus (DM) and affects 1 in 3 persons with DM [3]**.** Evidence showed that DR is responsible for 4.8% of the 37 million cases of blindness due to eye diseases throughout the world [4]. A follow up study revealed that 79.3% of individuals with type 1 and 82.6% of type 2 diabetes had evidence of ever having had a DR [5].

A Global meta-analysis study done in US, Australia, Europe and Asia reported that 1 in 3 (34.6%) had any form of diabetic retinopathy [6]. In the 2010 world diabetes population, more than 92 million adults had any form of Diabetic Retinopathy and DR develops with time and is associated with poor control of blood sugar, blood pressure, and blood lipids [3].

WHO reported that a good controller of diabetes and hypertension significantly reduces the risk for diabetic retinopathy [7]. Major risk factors of the DR may include the length of time the patient lived with diabetes, hyperglycemia, high blood pressure, lipid disorders, pregnancy and puberty, age at diagnosis, ethnicity and genetic disorders among others. DR develops as a consequence of the long-term accumulation of damage of retinal vessels [1]. Significant systemic risk factors include hypertension and high hemoglobin, systolic blood pressure (SBP), pulse pressure, serum lipoprotein level and body mass index (BMI). Other documented risk factors are renal disease/nephropathy, genetic factors, high waist-hip ratio (abdominal obesity), upper socioeconomic status, urban residence, male gender, insulin treatment and pregnancy [8].

In Ethiopia, around 2.1 million people are suffering from diabetes, of which 4.8% of adults are living with diabetes and 39% of these 2.1 million people are additionally suffering from DR [9]. From review of the existing evidences, determinants of Diabetic Retinopathy among diabetic patients on follow up in the Ethiopian health care setups have not been well documented.. Moreover, limited studies conducted in Ethiopia were cross-sectional studies; as a result, major risk factors were not well documented to guide interventions to reduce the new occurrence and target risk factors of DR. Therefore, this study was aimed to identify determinants of Diabetic Retinopathy in Jimma University Medical Center in Southwest Ethiopia.

## Methods

### Study setting and population

A facility based case control study was conducted in the Jimma University Medical Center (JUMC), the specialized hospital in Southwest of Ethiopia. The study was conducted from March 10 to May 09, 2018. The Hospital is located in Jimma City, (located 335 km Southwest of Addis Ababa, the capital). The hospital gives health services for more than 10 million people living in Southwest Ethiopia. The hospital has chronic follow-up clinics for both pediatric and adult patients. The diabetes clinic runs twice weekly and provides integrated diabetic care for both Type 1 and Type 2 diabetics for about 70–90 patients per day. In the hospital, there were around 3020 diabetic patients who follow regularly their treatments of which 946 patients were with DR and around 1605 of the patients were free of DR.

In this study, a case was defined as a diabetic patient with any of the following characteristic lesion: micro aneurisms, or hemorrhage, or hard exudates, or venous beading and new vessels on retina (diagnosis of new vessels on retina is a confirmatory tool for physicians or ophthalmologists that a DM patient has DR as a complication while control was defined as diabetic patients free of any of the criteria mentioned for cases in the above statement [10, 11]. The source population of the cases was all patients with DM and diagnosed with DR and visiting JUMC while the source population for controls was all patients with DM patients free of DR and visiting JUMC. All adults (above 15 years) DM patients who had no severe mental problems participated in the study while DM patients who were not volunteer to participate were excluded from the study.

### Sample size and sampling technique

Sample size was determined using EpiInfo statistical software version 7 using the following assumptions of two population proportion formula for the unmatched case-control studies: high baseline diastolic blood pressure (was taken as main exposure variable as it gave maximum sample size compared to other exposure variables) [12], 5% level of significance, 80% power, 2:1 control to case ratio, 9.47% expected prevalence of high baseline diastolic blood pressure among patients free of DR retinopathy (controls) and 21.1% expected prevalence of high baseline diastolic blood pressure among patients with DR (cases). As a result, 318 (106 cases and 212 controls) were considered to participate in the main study after considering 5% for non-response. Before the main study, cases (*n* = 946) and controls (*n* = 1605) were identified using the aforementioned case and control definitions. The sampling frame (list) of cases and controls was prepared systematic random sampling technique was used to select cases and controls for final data collection. Cases were selected from diabetic retinopathy patients and controls from free of retinopathy diabetic patients by systematic random sampling from ever enrolled diabetic patients on diabetic follow-up as a sampling frame. The kth value in the systmetaic sampling procedure for both case and control was calculated by diving each source population by the total sample size. For control the kth value was 5 and the 4rth patient was selected by using lottery method, then data was collected from every 5th patients starting from the 4th patient free of diabetic retinopathy by systematic random sampling. Similarly the kth value for the case was 3 from the list of 1 to 3 DM patient, the 2nd patient was selected by using lottery method, then data was collected from every 3rd patients starting from the 2nd patient with diabetic retinopathy by systematic random sampling.

### Data collection procedures

An interviewer-administered questionnaire was used to collect data from participants using the local language (Afan Oromo language). The questionnaire was developed after reviewing different kinds of literature [7, 12–17] (Additional file 1). The consistency of the questionnaire prepared in English translated into the local language (Afan Oromo) and back translating to English to check the consistency. Several medical-related data like blood pressure, cholesterol and glucose level, micro-vascular complications and hemoglobin level were collected from a patient’s record while socio-demographic, behavioral and other medical related data were collected by interviewing patients. Two trained Bachelor degree holders nurses collected the data. One Public Health degree holder supervised the data collection process. The quality of data was maintained by conducting the following: data collectors were trained for one day on how to collect data from participants, the whole contents of the questionnaire were explained to eliminate the difference between data collectors in understanding questions and pre-test was also conducted among 16 DM patients (5% of the sample size) in hospital outside Jimma town to ensure that the data collectors and respondents understood the questions. Accordingly, appropriate amendments were made on the questionnaire after pre-test. The data collection process was supervised and filled questionnaires were cross-checked for completeness and consistency. Finally, data were edited for possible errors, double entered using Epi-Data version 4.1 to eliminate errors that occur during data entry, and cleaned for missing values and outliers in SPSS version 20.

### Measurements

*Diabetic retinopathy* was diagnosed by ophthalmologists using slit lamp by dilating fundus examination using 1% tropic amide eye drop and diabetic patients who were considered to have damaged retinal blood vessels diagnosed and confirmed as diabetic retinopathy [18]. *Weight* was measured in light closing and without shoes in kilograms (kg) using calibrated digital weighing scale at a precision of 0.1 kg as per recommended but not repeated measurements. *Height* was measured using a stadiometer in centimeter (cm) in an erect position that the back of the head, shoulder blades, buttocks, and heels make a contact with the backboard at a precision of 0.1 cm with shoes removed as per recommended but not repeated measurements. *Blood pressure* was measured using a mercury sphygmomanometer with a cuff deflation rate of 2 mmHg. Two measurements were taken from left arm while the participant was in sitting position. Five minutes between each measurement was maintained to take avoid measurment bias. *Cholesterol level* was taken from the patient’s folder near to the time of data collection and if not present in the folder, 4 ml of the whole blood sample was taken and sent to the laboratory for both cases and controls. *Hemoglobin* was taken from the patient’s folder near to the time of data collection and if not present in the folder, 1 ml of the whole blood sample was taken and sent to the laboratory for both cases and controls. Operational definitions. *Adherence to medication* was defined as **“**if the patients took all his/her anti-diabetic medication in last seven days before the study” while *adherence to blood glucose testing at home* was defined as “if the patient measured his blood glucose for at least once every week [19, 20]”. *Alcohol consumption history* was defined as “if the patient reported consumption of any type of alcoholic beverages within the past 12 months were considered as alcohol consumers [20]”. *Glycemic control* was measured by random blood sugar. Average of the random blood sugar level of the last three records was used to define glycemic control. Any diabetic patient whose blood glucose measurement above 200 mg/dl was considered as good glycemic control while any diabetic patient whose blood glucose measurement 200 mg/dl and below was considered as poor glycemic control. *Adherence to diet* was assessed from report of particiapant wether he/she has followed recommended diet. If the patient followed the recommended diet forr more than 3 days in last seven days before the study period, that participant was labeled as adherent otherwise not adherent.

### Data analysis

The collected data were edited, coded and entered into Epi-Data version 4.1 and then exported to SPSS 20 for analysis. Descriptive statistics (frequencies, cross-tabulations and summary measures) were computed. Exposure variables associated with DR were identified using bivariate logistic regression analysis. Exposure variables with *p*-value < 0.25 were considered for final multivariable logistic regression analysis using the Backward Likelihood ratio method. Crude Odds ratios (CORs) and Adjusted Odds ratios (AOR) with their respective 95% CIs were computed for each exposure variable. *P*-value *<* 0.05was used to declare statistical significance and exposure variables with *p*-value < 0.05 were considered as determinants of DR. Model goodness of fit was checked using the Hosmer Lemeshow test of goodness of fit (x^2^ = 14.047, P-value= > 0.05(0.081), omnibus likelihood test *<* 0.05(0.000) with 89% model accuracy.

## Results

### Socio demographic characteristics

A total of 311 (97.8%) (106 cases and 205 controls) of DM patients who were on follow up in the Jimma University medical center participated in the study. The mean age (±Standard deviation) for the cases and the controls were 59.08 (SD: ±9.25) and 42.42 (SD: ±13.95) respectively. About two third, 65% of the cases and 60.9% of controls, were male participants. More than half, 52% of cases and 56.6% of controls were from rural residential. The majority of the cases (59.4%) and most of the controls (87.8%) were literate. Of those cases, 27.4 and 72.6% were government employees and self and private businesses respectively, but from the controls 20.5 and 79.5% were government employer and self and private business respectively (Table 1).

**Table 1:** Socio demographic characteristics of diabetic patients who follow at Jimma University Medical Center, Ethiopia, 2018

Variable	Category	Cases (***n*** = 106)	Controls (***n*** = 205)	COR (95%CI)	P-Value
Age	< 60 years ≥60 years	48 (45.3%) 58 (54.7%)	177 (86.3%) 28 (13.7%)	1 7.63 (4.39,13.27)	< 0.001
Residence	Urban Rural	51 (48%) 55 (52%)	89 (43.4%) 116 (56.6%)	1 0.82 (0.51,1.32)	0.43
Education level	Illiterate Literate	43 (40.6%) 63 (59.4%)	25 (12.2%) 180 (87.8%)	4.91 (2.77, 8.69) 1	< 0.001
Occupation	Government employee Self and private business	29 (27.4%) 77 (72.6%)	42 (20.5%) 163 (79.5%)	1 0.68 (0.39,1.18)	0.173
Family’s Monthly Income	<3061ETB ≥3061ETB	76 (71.7%) 30 (28.3%)	117 (57.1%) 88 (42.9%)	1.90 (1.15,3.15) 1	0.012
Sex	Male Female	69 (65%) 37 (35%)	125 (60.9%) 80 (39.1%)	0.83 (0.51,1.36) 1	0.477
Marital status	In Union Not in Union	101 (95.2%) 5 (4.8%)	152 (74.1%) 53 (25.9%)	7.04 (2.72,18.22) 1	< 0.001
Religion	Christians Muslim	39 (36.8%) 67 (63 .2%)	104 (50.7%) 101 (49.3%)	1.76 (1.09,2.86) 1	0.02
Ethnicity	Oromo Amhara Others	75 (70.7%) 22 (20.8%) 9 (8.5%)	127 (61.9%) 42 (20.5%) 36 (17.6%)	1 0.88 (0.49,1.59) 0.42 (0.19,0.92	0.690 0.032

*COR* Crude Odds Ratio, *CI* Confidence Interval, 1: reference group

### Behavioral characteristics

More than half, 55.6%, of cases and about two third, 63.4%, of controls were adhered to have regular exercise, and 38.6% of the cases and 55.1% of the controls adhered to the medication of diabetes. History of alcohol consumption was assessed and 15% of cases and 4.8%controls consumed the alcohol. Similarly, five of the participants from cases and two from the controls had a history of smoking. Regarding meal instruction adherence, about one third (35.8%) of cases and 62.9% controls adhered to meal instructions. From all study participants, 62 (58.5%) of cases and 166 (80.9%) of controls adhered to blood glucose measurement at home (Table 2).

**Table 2:** Behavioral characteristics of diabetic patients who follow at Jimma University medical center, Ethiopia, 2018

Variable	Category	Cases (*n* = 106)	Controls (*n* = 205)	COR(95% CI)	*P*-Value
Adherence to exercise	Yes No	59 (55.6%) 47 (44.4%)	130 (63.4%) 75 (36.6%)	1 1.38 (0.85,2.22)	0.185
Alcoholic history	Yes No	16 (15%) 90 (85%)	10 (4.8%) 195 (95.2%)	3.46 (1.51,7.93) 1	0.003
Smoking history	Yes No	5 (4.7%) 101 (95.3%)	2 (0.9%) 203 (99.1%)	5.02 (0.95,26.35) 1	0.056
Adherence to medication	Yes No	41 (38.6%) 65 (61.4%)	113 (55.1%) 92 (44.9%)	1 1.94 (1.20,3.14)	0.006
Adherence to meal	Yes No	38 (35.8%) 68 (64.2%)	129 (62.9%) 76 (37.1%)	1 3.03 (1.86,4.94)	< 0.001
Adherence to blood glucose	Yes No	62 (58.5%) 44 (41.5%)	166 (80.9%) 39 (19.1%)	1 3.02 (1.79,5.08)	< 0.001

*COR*: Crude Odds Ratio, CI: Confidence Interval, 1: reference group

### Medical history

More than nine-tenths (93.4%) of the cases and more than half (60.5%) of the controls were type II diabetic patients. There was a family history of DM in 56 (52.8%) of cases and 64 (31.2%) of controls. About half (48.1%) of the cases and less than one tenth (9.7%) of controls had a history or diagnosed as having other micro-vascular complications like heart and renal diseases. In this study, 41 (38.7%) of the cases and about one-tenth (9.3%) of controls had systolic hypertension, whereas 49 (46.3%) of cases and about one fourth (23%) of controls had diastolic hypertension. The proportion of poor glycaemic control was 63.2% in cases and 20% in controls. More than half (56.2%) of cases and more than one fourth (27.1%) of controls had also poor serum cholesterol levels. One third (32.4%) of cases and 8.7% of controls were anemic while 30 (28.3%) of cases and 59 (28.8%) of controls were overweight (Table 3).

**Table 3:** Medical history of diabetic patients who follow at Jimma University medical center, Ethiopia, 2018

Variable	Category	Cases (*n* = 106)	Control (*n* = 205)	COR(95% CI)	*P*-Value
Family history of DM	Yes No	56 (52.8%) 50 (47.2%)	64 (31.2%) 141 (68.8%)	2.46 (1.52,3.99) 1	< 0.001
Duration of DM after diagnosis	< 6 years ≥6 years	36 (33.9%) 70 (66.1%)	142 (69.2%) 63 (30.8%)	1 4.38 (2.65,7.22)	< 0.001
Other micro-vascular complications	Yes No	51 (48.1%) 55 (51.9%)	20 (9.7%) 185 (90.3%)	8.57 (4.71,15.60) 1	< 0.001
Systolic blood pressure	< 140 mmHg ≥140mmhg	65 (61.3%) 41 (38.7%)	186 (90.7%) 19 (9.3%)	1 6.17 (3.34,11.39)	< 0.001
Diastolic blood pressure	<90mmhg ≥90mmhg	57 (53.7%) 49 (46.3%)	158 (77%) 47 (23%)	1 2.89 (1.75,4.77)	< 0.001
Glycaemic level (RBS)	Good Poor	39 (36.8%) 67 (63.2%)	164 (80%) 41 (20%)	1 6.87 (4.07,11.58)	< 0.001
Serum Cholesterol level	Good Poor	54 (56.2%) 42 (43.8%)	51 (27.1%) 137 (72.9%)	1 0.29 (0.17,0.48)	< 0.001
Haemoglobin level	< 11 mg/dl ≥11 mg/ml	34 (32.4%) 71 (67.6%)	18 (8.7%) 187 (91.3%)	4.97 (2.64,9.37) 1	< 0.001
BMI	< 25 kg/m^2^ ≥25 kg/m^2^	76 (71.7%) 30 (28.3%)	146 (71.2%) 59 (28.8%)	1 0.97 (0.58,1.64)	0.929

### Determinants of diabetic retinopathy

In a multiple logistic regression analysis; age 60 years and above, lower educational level (illiterate), poor adherence to medication, family history of Diabetes Mellitus, presence of other micro vascular complication, poor glycemic control, systolic hypertension, poor cholesterol level and being anemic patients were significantly associated with the development of diabetic retinopathy.

The odds of developing Diabetic retinopathy were almost five times higher in patients with age 60 years and above than patients under 60 years of age (AOR = 5.04: 95%CI; 1.83, 13.87). The study revealed that illiterate diabetic patients had about seven times higher odds of developing diabetic retinopathy than literates (AOR = 7.17, 95%: CI 2.61, 19.70). Patients who did not adhere to medication were three times more likely to develop DR than diabetic patients who did not adhere to medication (AOR = 3; 95%CI: 1.29, 6.95). The study also revealed that the odds of developing DR were more than three times higher for patients with baseline Systolic Blood Pressure level of 140 mmHg and above than their counters parts (AOR: 3.38, 95%CI: 1.26, 9.05). The odds of developing DR were nine times higher in diabetic patients with poor blood glucose control than diabetic patients in good blood glucose control (AOR: 9.08, 95%CI: 3.7, 22.29).

The study also revealed that participants who had a family history of DM and who had other micro-vascular complications were about four times higher odds of developing DR than their counterparts (AOR = 3.95; 95%CI: 1.64, 9.54), (AOR = 3.76; 95%CI: 1.33, 10.66) respectively. In the study, patients with poor cholesterol levels were about 79% times less likely to develop DR than diabetic patients who had a good serum cholesterol level (AOR = 0.21, 95%CI: 0.08, 0.514). Diabetic patients who were anemic were more than two and half have times the odds of developing DR than their counterparts (AOR = 2.8, 95%CI: 1.05,7.47) (Table 4).

**Table 4:** Multivariate logistic regression analysis of Diabetic Retinopathy in Jimma University Medical Center, Ethiopia, 2018

Variable	Category	Cases (*n* = 106)	Control (*n* = 205)	COR (95%CI)	AOR(95% CI)	*P*-Value
Age	< 60 years ≥60 years	48 (45.3%) 58 (54.7%)	177 (86.3%) 28 (13.7%)	1 7.63 (4.39,13.27)	1 5.04 (1.83,13.87)	0.002
Education level	Illiterate Literate	43 (40.6%) 63 (59.4%)	25 (12.2%) 180 (87.8%)	4.91 (2.77, 8.69) 1	7.17 (2.61,19.70) 1	< 0.001
Adherence to medication	Yes No	41 (38.6%) 65 (61.4%)	113 (55.1%) 92 (44.9%)	1 1.94 (1.20,3.14)	1 3 (1.29,6.95)	0.01
Family history of DM	Yes No	56 (52.8%) 50 (47.2%)	64 (31.2%) 141 (68.8%)	2.46 (1.52,3.99) 1	3.95 (1.64,9.54) 1	0.002
Other micro-vascular complications	Yes No	51 (48.1%) 55 (51.9%)	20 (9.7%) 185 (90.3%)	8.57 (4.71,15.60) 1	3.76 (1.33,10.66) 1	0.013
Systolic blood pressure	<140mmhg ≥140mmhg	65 (61.3%) 41 (38.7%)	186 (90.7%) 19 (9.3%)	1 6.17 (3.34,11.39)	1 3.38 (1.26,9.05)	0.015
Glycaemic level (RBS)	Good Poor	39 (36.8%) 67 (63.2%)	164 (80%) 41 (20%)	1 6.87 (4.07,11.58)	1 9.08 (3.70,22.29)	< 0.001
Serum Cholesterol level	Good Poor	54 (56.2%) 42 (43.8%)	51 (27.1%) 137 (72.9%)	1 0.29 (0.17,0.48)	1 0.21 (0.08,0.514)	0.001
Haemoglobin level	< 11 mg/dl ≥11 mg/ml	34 (32.4%) 71 (67.6%)	18 (8.7%) 187 (91.3%)	4.97 (2.64,9.37) 1	2.8 (1.05,7.47) 1	0.038

## Discussion

This study revealed that participants whose age were 60 years and above were five times odds of developing diabetic retinopathy when compared with patients whose age was less than 60 years. Similar findings were reported in various countries. According to the cohort study done in Arbaminch General hospital [13], the possibility of developing DR was almost seven times higher among patients who were 60 and above years than their counterparts. A study from England [5], Armenia [21] and United States [22] also showed that older age was strongly associated with the occurrence of diabetic retinopathy. The study from Oman also indicated that the retinopathy rate was higher in age 60 years and above than their counterparts [23]. As being older age was a strong risk factor for DR, developing any retinopathy was higher in older age [15]. The primary reason for this is that many elderly diabetic patients suffer from physical and mental ailments and often take poor control of their blood sugar especially in consideration of their social background [24].

Illiterate diabetic patients had seven times higher odds of developing DR than literate patients. Studies conducted in Saudi Arabia [25], Korea [26] and Sudan [18] suggest that less educated or who had no formal education had a high probability of developing DR than who attained higher classes. Another finding from Tokyo suggests that lack of proper educational attainment was significantly associated with the progression of retinopathy [27]. The study done in Korean population revealed that illiterate patients had a high probability of developing diabetic complications like DR [28]. Providing proper health information and counseling on the consequences of poor adherence to diabetic care improves the clinical outcomes of diabetic patients [29].

A diabetic who were poorly adherent to diabetic medication was three times odds of DR than diabetic patients who were good adherent. This finding is consistent with studies done in rural India, Armenia and Sudan as poor adherence to medication had significant association with the development of diabetic retinopathy [18, 19, 30].

This study showed that the odds of developing DR were higher in diabetic patients with a family history of diabetes. This is similar to studies conducted in Arbaminch [13] and China [31]. Finding from Southwest of Ethiopia showed diabetic patients who had family history diabetes were three-times more susceptible to the development of micro vascular complications like DR [12]. Diabetic patients who had poor glycemic control had higher odds of developing retinopathy than those with good glycemic control. Similarly, the studies done in Arbaminch [13], Saudi Arabia [10], China [32], Singapore [33], and Qatar [34] also reported as Diabetic patients with poor glycemic control had a high probability of developing diabetic retinopathy.

This study revealed poor cholesterol control was negatively associated with the occurrence of DR. This finding is similar with the study done in Singapore which reported that higher cholesterol levels were protective of any retinopathy [35]. However, the study done in Saudi Arabia [10], China [15] and Malawi [36] revealed that poor control of cholesterol increases the probability of DR their counterparts.

In this study diabetic patients who were with systolic hypertension (Systolic Blood Pressure greater than 140 mm hg) was also one independent factor that determines the development of DR among diabetic patients. Similar findings were also reported in China [15], Arbaminch [14], Korea [28] and in rural India [37].

This study revealed the presence of other micro vascular complication was another determinant of diabetic retinopathy. The finding is consistent with the study done in high-risk China population [30] that revealed renal problem was one of the risk factors associated with DR. Therefore, patients who had history or diagnosed as having any other micro vascular complication like diabetic nephropathy and cardiac diseases had higher odds of developing diabetic retinopathy [28]. Being anemic had also contributed to the occurrence of diabetic retinopathy among diabetic patients. Similar studies in Singapore [33], San Franscisco [38] and India [29] indicated that diabetic patients who were anemic was independently associated with an increased risk of developing DR. But cohort study done in china [15], Korea [28] and Ethiopia [12] suggested that higher hemoglobin was independently associated with an increased risk of DR. These differences could be anemia induced retinal hypoxia which alters angiogenesis, capillary permeability, vasomotor response, and cell survival and also due to low plasma ferritin concentration [11]. Those researchers who considered high hemoglobin leads DR considers that diabetic patients with poor glycemic control have high hemoglobin (plasma ferritin concentration) significantly predicted major micro vascular complications like DR which in turn damages retinal blood vessels [12].

The limitations of this study were behavioral variables like adherence to diabetic care, blood pressure, cholesterol, and hemoglobin level, were collected from patient records. These data may not show the measurements prior to the development of DR. On the other hand, the possibility of information bias especially on alcohol consumption history, smoking history, adherence to diabetic care and wide confidence of random blood glucose control could affect the validity of and precision of the study. Even though measurement for weight and height were taken by health professionals, a single measurement may not be accurate, measurement bias could affect this study.

## Conclusion

The findings in this study shows that older age of 60 years and above, lack of proper educational, poor adherence to anti DM medication, being born from having family history of DM, presence of other microvascular complication like renal problem and heart diseases, poor blood glycemic control level, having systolic hypertension, poor serum cholesterol level and being anemic patients were significantly associated with the development of diabetic retinopathy. Therefore, there is a need to give attention by supplying necessary logistics, assigning trained health professionals and necessary training for who lacks proper trainings for different health professionals who could treat diabetic patients. Similarly, there is a need to consider older age patients, and creating good awareness to have optimal adherence to medication, especially for illiterate patients. Moreover, early identification and prompt management of patients with other micro-vascular complications are vital. Patients adherence to physicians’ advice and provided medication is also crucial.

## Supplementary information

**Additional file 1.** Questionnaire to assess determinants of Diabetic Retinopathy

## Data Availability

Data will be available upon request from the corresponding author.

## Abbreviations

AOR: Adjusted Odds Ratio
BMI: Body Mass Index
BP: Blood Pressure
DBP: Diastolic Blood Pressure
DM: Diabetic Mellitus
DR: Diabetic Retinopathy
FBS: Fasting Blood Sugar
IGR: Impaired Glucose Regulation
SBP: Systolic Blood Pressure
