# Supplementary material for: Determinants of diabetic retinopathy in Southwest Ethiopia: a facility-based case-control study
Source: BMC Public Health. 2020 Apr 15;20:503. doi: 10.1186/s12889-020-08652-2 (PMC7161237; doi:10.1186/s12889-020-08652-2)
Supplement: Supplementary file 1 — Additional file 1. Questionnaire to assess determinants of Diabetic Retinopathy [file 12889_2020_8652_MOESM1_ESM.docx]

**Additional file 1: Questionnaire to assess determinants of Diabetic Retinopathy**

Part 1: socio economic /demographic conditions

| s.no | Questions | Response | Skip |
| --- | --- | --- | --- |
| Q101 | Age | --------------------years |  |
| Q102 | Sex | 1.Male 2. Female |  |
| Q103 | Residence | 1.urban 2. rural |  |
| Q104 | Marital status | 1.Married  2.Single  3. Widowed  4. Divorced |  |
| Q105 | Educational level | 1. cannot read and write 2. can read and write 3. Primary school (1-8 ) 4. secondary school (9-12) 5. diploma 6. degree and above |  |
| Q106 | Occupational status | 1.student  2.merchant  3. farmer  4.government employee  5.self employed  6.house wife  7.others, specify------ |  |
| Q107 | Religion | 1.protestant  2.orthodox  3.catholic  4.muslim  5.others , specify----------- |  |
| Q108 | Ethnicity | 1. Oromo  2.Amhara  3.Tigre  4.Gurage 5.Other(Specify)___________ |  |
| Q109 | Income (in cash) | -----------------------in Ethiopian birr |  |

Part 2 – Patient’s Behavioral and life style factors related questions **(Patient-Interview)**

| Q no | Question | response | skip |
| --- | --- | --- | --- |
| Q201 | Do you have exercise plan you set with your doctor (like fast walking, dancing )? | 1.yes  2.No | If no go to Qno 203 |
| Q202 | If yes to Q.no 201 How many days a week do you do some form of moderate exercise (like fast walking)? | -------------days/week |  |
| Q203 | Have you consumed alcohol (within the last 12 months)? | 1.yes 2.no | If no Skip to No.206 |
| Q204 | In the past 12 months, how frequently have you had at least one drink? | ________(daily/amounts per week or month) |  |
| Q205 | When you drink alcohol, on average, how many drinks do you have during one day? | Number ___ __   Don't know |  |
| Q206. | Have you ever smoked? | 1.yes 2. No | If no, go to Q209 |
| Q207. | If yes, do you smoke now? | 1.yes 2. No |  |
| Q208. | \| If yes Qno. 207 , How many packets per day do you smoke when you smoke? \| \| --- \| | _____________ |  |
| Q209 | How many times a day, are you should to take medications to lower your blood sugar? | 1. Once a day 2. twice a day 3. three times or more |  |
| Q210 | How many times a week you did not take your medication for blood sugar? | ______ (a number) | Enter “0 “ for no |
| Q211 | For Qno. 210 , Which one of the following was the reason for not taking your medication for blood sugar? | 1.Cost of medication too expensive  2.Forgetfulness  3.Feeling well without medications  4. Complex regimen  5.Physicians mode of approach  6. Lack of trust on the efficacy of medications  7.othrs, specify (___________) |  |
| Q212 | Did you have meal plan? | 1. Yes 2. no | If no skip to Qno.215 |
| Q213. | If yes what type of meal plan have you decided to follow? | 1.small frequent meals  2. five or more fruits and vegetables a day  3. counting carbohydrates  4. other (please specify) _________________ |  |
| Q214 | In the last week, how many days of the week did you follow your meal plan? | ___________ (To follow it, you would have had to eat all 3 meals that day according to your chosen meal plan). | Enter “0 “ for no |
| Q215 | Do you have regular glucose measurement? | 1.yes 2. no |  |
| Q216 | How many times a week do you check your blood sugar? | _______ | Enter “0 “ for no |
| Q217 | Do you have Family history of diabetes? | 1.Yes 2. No |  |

Part 3- Patients data to be filled from patient chart and/or to be measured by the interviewer.

| **QNO.** | **QUESTIONS** | **RESPONSE** | **SKIP** |
| --- | --- | --- | --- |
| Q301 | Type of DM | type 1 2. type 2 |  |
| Q302 | duration of diabetes after diagnosis | _____________years/months (write in months if<1year). |  |
| Q303 | presence of diabetic retinopathy (DR)(from patient chart) | 1.Yes 2. no | If no skip to Qn No.305 |
| Q304 | If yes to Qn 303 which type of DR | 1. Proliferative DR 2. Non-proliferative DR |  |
| Q305 | Medication the patient was on (from patient chart) | 1.oral hypoglycemic agent  2.insulin  3.oral hypoglycemic agents and insulin  4.not on any medication |  |
| Q306 | Specific medication the patient was on (from patient chart) | 1. NPH insulin  2.Glibenclamide and metformin  3.Metformin  4.Glibenclamide  5. Metformin and NPH insulin |  |
| Q307 | presence of micro-vascular complications other than retinopathy that was on the patient card) | 1. Yes 2. No | If no skip to no. 309 |
| Q308 | If yes to Qn no.307 encircle from options more than one is possible | 1. Cardio-vascular 2. Renal disease 3. No ,any of the above complications |  |
| Q309 | Blood pressure (near to data collection) (measured by data collectors). | SBP____________mmHg  DBP____________mmhg |  |
| 310 | Fasting blood sugar (recent 3 measurements). | Reading 1__________mg/dl  Reading 2_______ ___mg/dl  Reading 3____________mg/dl  Average _____________mg/dl |  |
| Q311 | Hemoglobin (from card or if done on data collection time). | ___________mg/dl |  |
| Q312 | Cholesterol level (from card or if done on data collection time) | _____________mg/dl |  |
| Q313 | (Weight &Height measured by data collectors) | Weight _______kg  Height ________cm, |  |
